# Biological Functions and Therapeutic Potential of Autophagy in Spinal Cord Injury

**DOI:** 10.3389/fcell.2021.761273

**Published:** 2021-12-20

**Authors:** Hai-Yang Liao, Zhi-Qiang Wang, Rui Ran, Kai-Sheng Zhou, Chun-Wei Ma, Hai-Hong Zhang

**Affiliations:** ^1^ Lanzhou University Second Hospital, Lanzhou, China; ^2^ Department of Orthopaedics, Lanzhou University Second Hospital, Lanzhou, China

**Keywords:** spinal cord injury, autophagy, autophagy-regulated, therapeutic target, biological functions

## Abstract

Autophagy is an evolutionarily conserved lysosomal degradation pathway that maintains metabolism and homeostasis by eliminating protein aggregates and damaged organelles. Many studies have reported that autophagy plays an important role in spinal cord injury (SCI). However, the spatiotemporal patterns of autophagy activation after traumatic SCI are contradictory. Most studies show that the activation of autophagy and inhibition of apoptosis have neuroprotective effects on traumatic SCI. However, reports demonstrate that autophagy is strongly associated with distal neuronal death and the impaired functional recovery following traumatic SCI. This article introduces SCI pathophysiology, the physiology and mechanism of autophagy, and our current review on its role in traumatic SCI. We also discuss the interaction between autophagy and apoptosis and the therapeutic effect of activating or inhibiting autophagy in promoting functional recovery. Thus, we aim to provide a theoretical basis for the biological therapy of SCI.

## 1 Introduction

Spinal cord injury (SCI) is one of the most important health problems globally, posing a huge challenge to clinical medicine and basic research and imposing a heavy burden on patients and society (Dumont et al., 2001; Gupta et al., 2010; Zhou et al., 2017a). Worldwide, an estimated 2.5 million people suffer from SCI, with more than 130,000 new injuries reported each year (Thuret et al., 2006). SCI is the most common disabling spinal injury, which can destroy the anatomical structure of the spinal cord, resulting in a series of pathological reactions such as axon rupture, neuron degeneration and necrosis, inflammatory response, and demyelination of the myelin sheath, eventually leading to severe neurological dysfunction (Elkabes and Nicot, 2014; Huang et al., 2017; Vismara et al., 2020). In addition, SCI often leads to sensorimotor disorders, autonomic nervous changes, and intractable pain. It may also affect respiratory, urinary, and gastrointestinal functions, and be a factor for developing an infection, thus seriously affecting patients’ quality of life (Dowler et al., 1997; Dumont et al., 2001; Erlich et al., 2007). To date, treatment options for SCI include conservative and surgical treatment. Conservative treatment aims to relieve pain, using opioids or local anesthetic injections, massage, and acupuncture (Saal and Saal, 1989; Wang et al., 2020). Surgical intervention involves mainly decompression of the spinal cord (Karsy and Hawryluk, 2019). Although conservative and surgical treatment may improve patient’s status, their complications and economic burden are considerable. Therefore, exploring the SCI pathological mechanisms and their implementation in biological therapy for the early recovery of the spinal cord neuron connectivity and neurological function is of great importance.

Autophagy is a general term for an evolutionarily conserved, lysosome-based degradation process playing an important role in various pathophysiological environments (Mizushima and Klionsky, 2007; Mizushima and Levine, 2010; Yang and Klionsky, 2010; Mizushima and Komatsu, 2011; Rubinsztein et al., 2012a; Russell et al., 2014; Avin-Wittenberg, 2019). These include starvation as an adaptive response, intracellular proteins and organelles quality control, anti-aging, tumor formation inhibition, and microorganisms’ elimination in cells (Mizushima and Klionsky, 2007; Levine and Kroemer, 2008; Mizushima et al., 2008; Deretic and Levine, 2009). Autophagy is an important defense and protective mechanism of the body. Autophagy is mainly involved in cell recycling by degrading and removing damaged and denatured proteins and lost organelles (Mizushima, 2007; Wirawan et al., 2012; Avin-Wittenberg, 2019). However, overinduction of autophagy can lead to autophagic cell death (Zhou et al., 2015). Research has shown that autophagy plays an important role in SCI. Most scholars believe that abnormal regulation of autophagy and abnormal nutritional composition are important recovery mechanisms after SCI (Li et al., 2019). We review the pathophysiological process of autophagy after SCI, focusing on cell biology, the activation and regulation processes, and the duality of autophagy. Recent findings on autophagy’s role in SCI, the regulation, and the level of autophagy flux may become a potential new neuroprotective target, providing a new idea for the clinical treatment of SCI.

## 2 The Pathophysiology of Spinal Cord Injury

**FIGURE 1:**
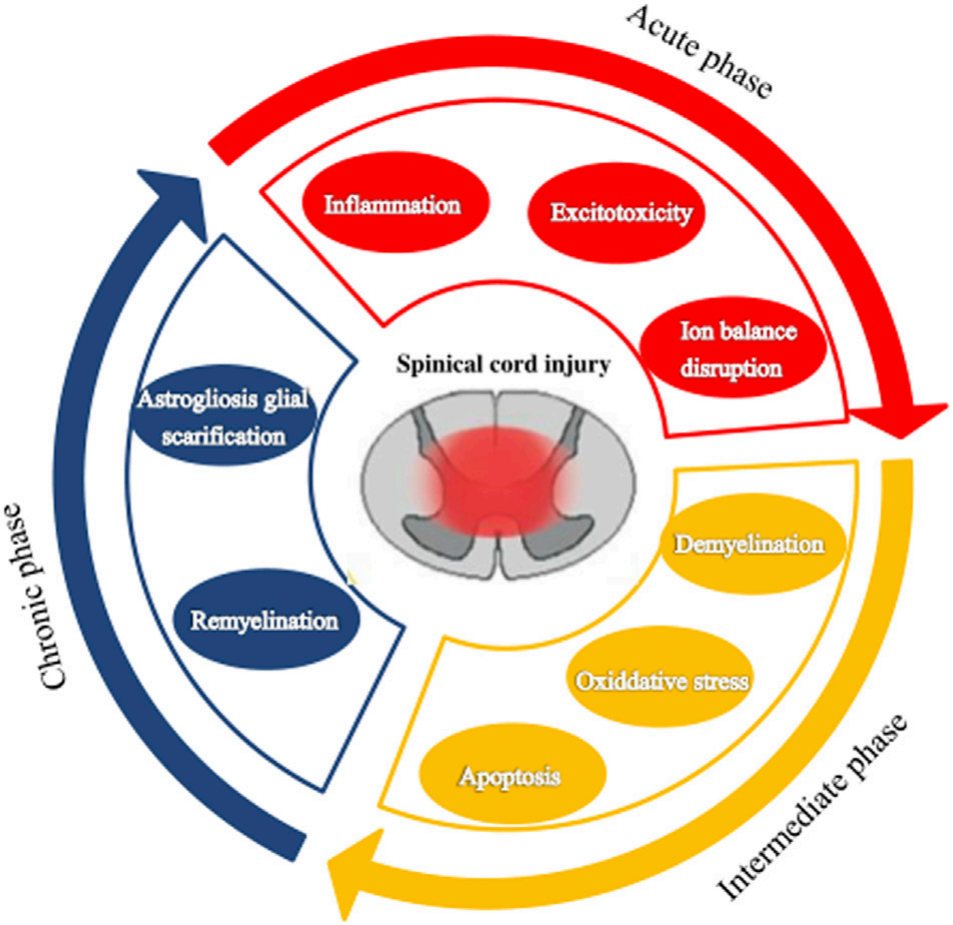
The pathophysiological processes following spinal cord injury. Spinal cord injury can be divided into three stages: acute, subacute (or moderate), and chronic.

According to the pathophysiological process, SCI can be divided into primary injury and secondary injury (Lukovic et al., 2015; Alonso-Calviño et al., 2016). However, in many clinical situations, secondary injury is more important as it is closely related to the prognosis of SCI and the therapeutic target is to prevent the spread of the injury (Rowland et al., 2008). Primary injury is a direct injury and necrosis of the spinal cord tissue and nerve cells caused by external mechanical forces (Lukovic et al., 2015). Secondary injury is a delayed (from a few hours to weeks) and progressive tissue injury following a primary injury. It presents with spinal cord hemorrhage, edema, ischemia, free oxygen damage, electrolyte disturbance, excessive release of excitatory toxins, and an inflammatory response leading to a large number of neuronal apoptosis, demyelination of residual nerve fibers, and a series of biological events (Kwon, 2004; Kim et al., 2017). After a mechanical injury, structural damage and vascular dysfunction lead to edema, necrosis, and ischemia, including bleeding, vasospasm, thrombosis, loss of autoregulation, and destruction of the blood-spinal barrier. In addition, this damage causes more inflammatory cells to cross the defective barrier, amplifying the initial injury. The secondary injury process can be divided into acute, subacute (or moderate), and chronic stages, based on the pathologic mechanism and elapsed time (Figure 1).

The acute phase is considered to last 48 h after the initial physical injury (Tator and Fehlings, 1991; Tator and Koyanagi, 1997; Kwon, 2004). It presents mainly with vascular rupture, hemorrhage, and ischemia. The microcirculation disruption is followed by ions dysregulation, excitotoxicity, excessive production of free radicals, and inflammatory response, which are associated with further damage of neurons and glial cells (Schanne et al., 1979; Ha et al., 2011). Excitotoxicity results from the overactivation of excitatory neurotransmitters (glutamate, aspartic acid), and the overexpression of these excitatory amino acids can induce apoptosis of neurons and glial cells especially oligodendrocytes (Li et al., 1999; Li and Stys, 2000; Park et al., 2004). Lipid peroxidation mediated by free radicals can lead to membrane damage, resulting in cell lysis, organelle dysfunction, and intracellular ion homeostasis disorders (Xiong et al., 2007; Donnelly and Popovich, 2008). Inflammation is thought to be an important mechanism of secondary injury. Macrophages, astrocytes, T cells, microglia, and other inflammatory cells infiltrate the lesion area and participate in the inflammatory response, releasing tumor necrosis factor-α (TNF-α), interleukin (IL)-1α, IL-1β, IL-6, and other inflammatory factors, leading to cell necrosis or apoptosis, and the generation of free radicals causing destruction of endothelial cells and loss of astrocyte function. This series of reactions disrupt the spinal cord microenvironment.

The subacute phase is considered to last up to 2 weeks after the injury. Astrocytes’ reactive proliferation characterizes this stage. Injured astrocytes would be activated to produce reactive cell proliferation, hypertrophy, and fibrosis and eventually form dense glial scars (Fawcett and Asher, 1999; Sofroniew and Vinters, 2010). Glial scars act as dense mechanical barriers that make it difficult for axons to cross and activate astrocytes to secrete some inhibitory extracellular matrix molecules, such as chondroitin sulfate proteoglycan, which act as a chemical barrier to inhibit the growth and regeneration of axons (Barritt et al., 2006; Koh et al., 2018). Thus, SCI regeneration is affected (Sofroniew, 2009; Karimi-Abdolrezaee and Billakanti, 2012). However, astrocytes are a “double-edged sword” in SCI (Karimi-Abdolrezaee and Billakanti, 2012; Okada et al., 2018). Astrohyperplasia can promote the healing of injured nerve tissue, recovery of microenvironmental homeostasis, and reconstruction of the integrity of the blood-brain barrier. On the other hand, as a barrier, it can reduce edema, limit the infiltration of immune cells, and limit the spread of inflammation from the injury to the surrounding areas. Thus, the injured surrounding tissues and cells could avoid further damage (Lukovic et al., 2015). Later, activated astrocytes secrete growth-promoting neurotrophic factors exerting endogenous neuroprotective effects (Chen et al., 2006), such as brain-derived neurotrophic factor, vascular endothelial growth factor (VEGF), and nerve and basic fibroblast growth factor. These growth factors promote the migration, proliferation, and differentiation of oligodendrocyte progenitor cells to prevent demyelination after injury.

The chronic phase is generally considered to start after 6 months of impairment, though this is controversial. Maturation of the lesion, including scar formation and development of a syrinx, is regarded as a characteristic of the chronic phase of SCI. Therefore, treatment focuses on enhancing the regeneration of the damaged axons and myelin sheath and preventing the formation of glial scars. But so far, there has been no significant effect.

## 3 An Overview of Autophagy

Autophagy is a conserved, closely coordinated self-degradation and cycling process that commonly occurs in eukaryotic cells and maintains metabolism and homeostasis *in vivo* (Parzych and Klionsky, 2014; Avin-Wittenberg, 2019). It isolates misfolded proteins, damaged or aged organelles, and mutated proteins in the double-membrane vesicles of autophagosomes. It fuses them into the lysosomes, leading to the degradation of the isolated components and recycling the decomposed macromolecules (Behrends et al., 2010). Autophagy is conserved from yeast to humans, regulates the homeostasis in physiological and pathophysiological environments, and is involved in the occurrence and development of various diseases. Autophagy has been identified as a therapeutic intervention target for a variety of diseases, including SCI.

### 3.1 Classification and Physiology of Autophagy

**FIGURE 2:**
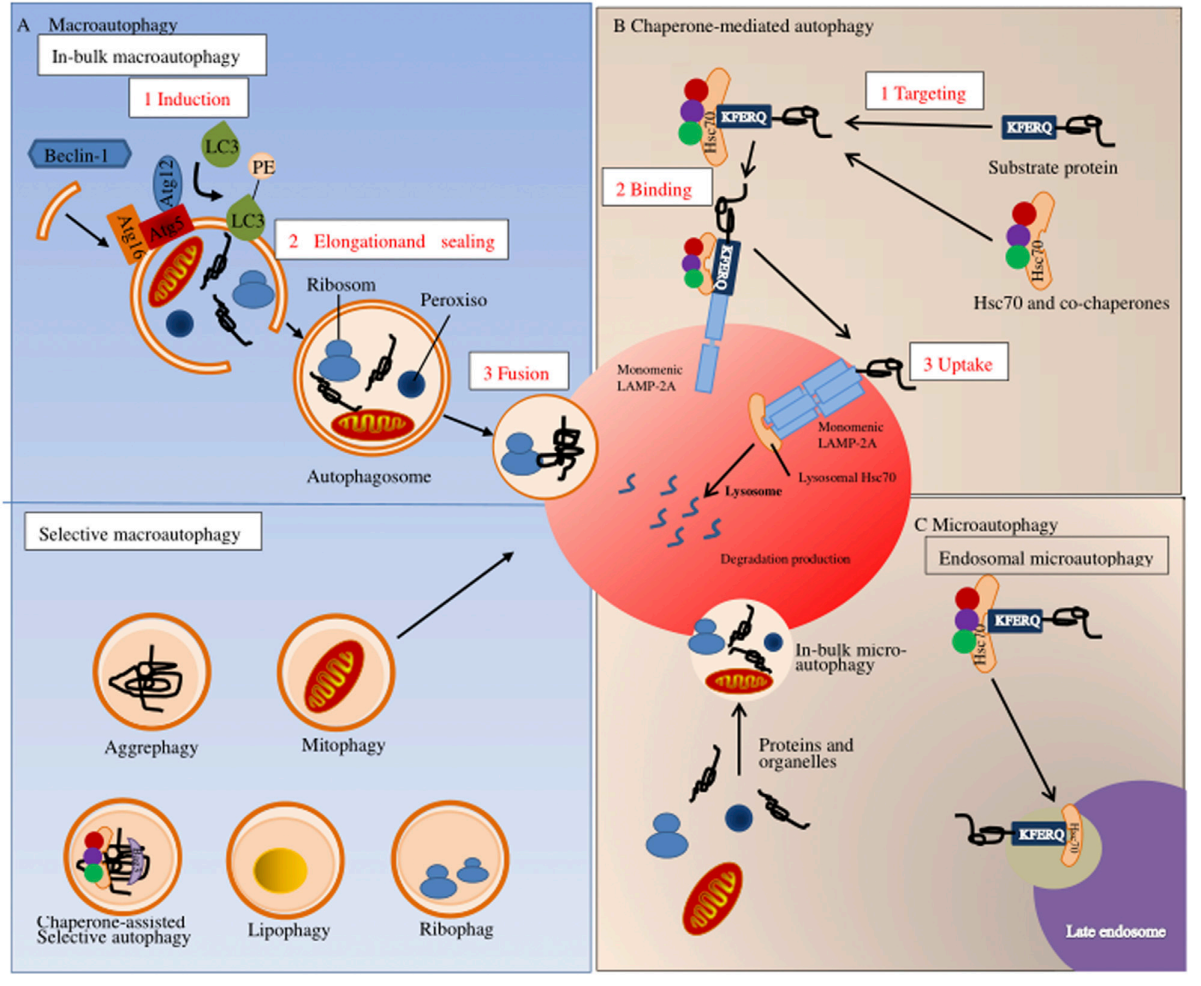
Autophagy pathways in mammals. Autophagy in mammals can be divided into three types: **(A)** macroscopic autophagy; **(B)** chaperone-mediated autophagy; **(C)** microautophagy.

Autophagy is a process of cell degradation and recycling. In eukaryotic cells, autophagy can be roughly divided into three categories: macroautophagy, microautophagy, and chaperone-mediated autophagy (Yang and Klionsky, 2010; Mizushima and Komatsu, 2011). Macro-autophagy is the main regulatory form of autophagy, participating in response to environmental and physiological cues. Microautophagy involves the direct lysosomal phagocytosis of cytoplasmic contents (Schuck, 2020). In contrast, chaperone-mediated autophagy consists of the translocation of chaperone-assisted substrate proteins [possibly deoxyribonucleic acid (DNA) and ribonucleic acid (RNA)] across lysosomal membranes (Fujiwara et al., 2017; Kaushik and Cuervo, 2018). In macroautophagy, the cytoplasmic cargo first isolates itself from the lysosome in a synthesized two-membrane vesicle, autophagosome. Then it is transported to the lysosome for complete degradation, where autophagy-related genes and autophagy-related proteins are involved (Bento et al., 2016; Levine and Klionsky, 2017). During microautophagy, the invaginations or protrusions of the lysosomal membrane are used to capture the cytoplasmic cargo, which is finally internalized through lysosomal and endosomal membrane invagination (Yorimitsu and Klionsky, 2005; Tekirdag and Cuervo, 2018). Furthermore, in chaperone-mediated autophagy, proteins are recognized by one cytoplasmic chaperone and brought to the lysosomal surface, where they are then translocated on the membrane (Kaushik and Cuervo, 2018) (Figure 2). Although the three types of autophagy are different in morphology and mechanism of action, they all eventually transport materials to lysosomes for degradation and recycling.

Autophagy maintains the body’s dynamic balance and regulates the health and functioning of cells through a variety of physiological mechanisms. A key physiological role of autophagy in cellular metabolism is mobilizing intracellular energy resources to meet the needs of cells and organisms (Kim and Lee, 2014). There are two main ways for cells to maintain their energy supply. The first is to obtain extracellular nutrients regulated by growth factor signaling pathways, provide oxidizable substrates to cells, and support adenosine triphosphate (ATP) production and biosynthesis through anabolism (Edinger et al., 2003; Barata et al., 2004). When the growth factor signaling pathway is weakened, followed by nutrient deprivation, growth factor consumption, hypoxia, and other conditions, autophagy degrades to release amino acids, which are further processed and used together with fatty acids in the tricarboxylic acid cycle for adaptive protein synthesis and maintenance of cell ATP production (Lum et al., 2005). The second basic function is intracellular quality control (Lim and Yue, 2015). The day-to-day management functions of autophagy include removing defective proteins and organelles, preventing abnormal protein aggregation, and removing intracellular pathogens. Related studies have found that in the case of autophagy deficiency, the circulation of cytoplasmic proteins is impaired, which increases their tendency to damage and misfold, followed by ubiquitination and aggregation (Hara et al., 2006; Komatsu et al., 2006). These functions may be critical for autophagy-mediated protection against aging, cancer, and infection. Finally, autophagy may act as a guardian of the genome. Given the known role of autophagy in energy homeostasis and quality control of proteins and organelles, as well as recent studies on immortalized epithelial cells with autophagy-related (ATG) gene defects, autophagy can limit DNA damage and chromosomal instability (Mathew et al., 2007). These physiological functions of autophagy allow the autophagy system to play an important role in the survival of organisms and be interrelated with various diseases.

### 3.2 The Process of Autophagy

**FIGURE 3:**
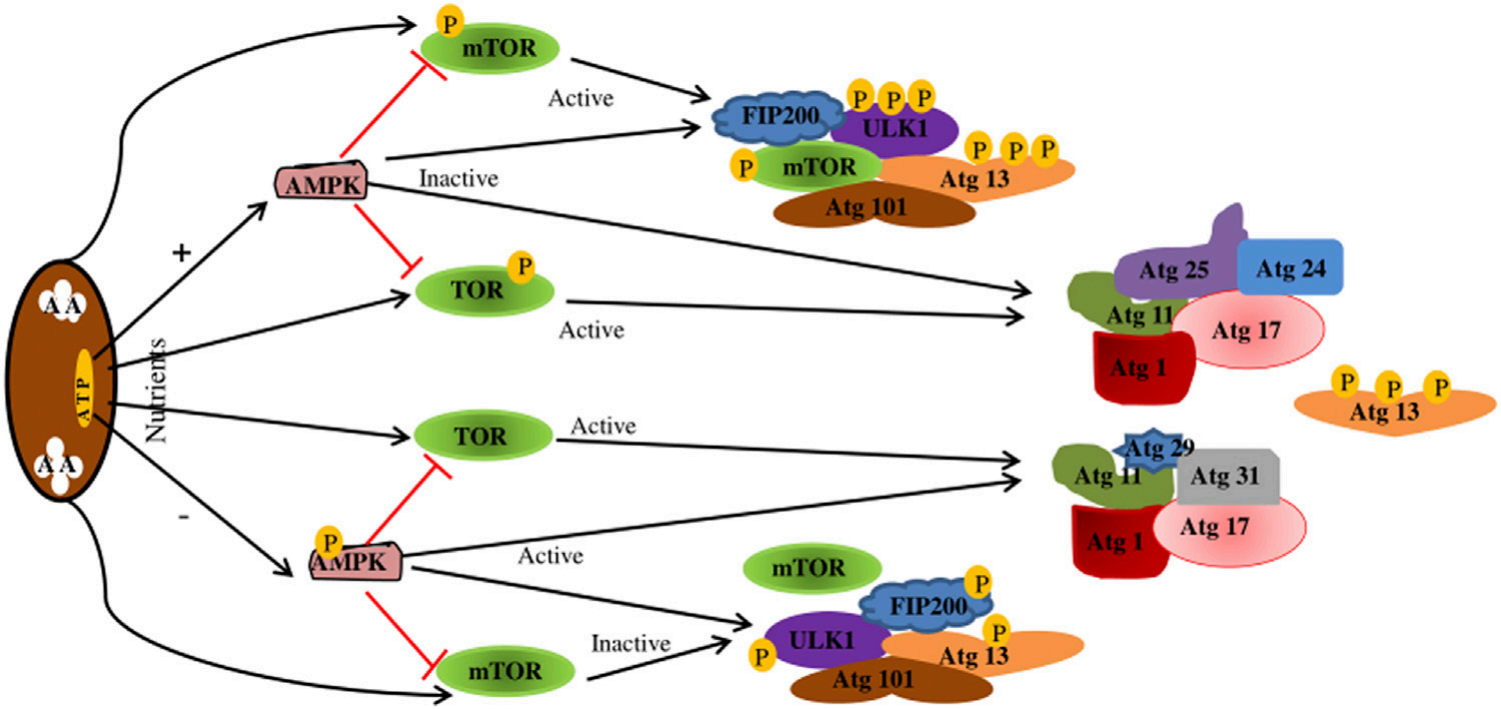
Regulation of the Atg1/ULK1 complex. When nutrition is sufficient, AMPK is inactivated, TOR is activated, Atg13 is phosphorylated, the kinase activity of Atg1 complex is reduced, and autophagy is inhibited. mTOR binds to ULK1SER757, inhibiting ULK1-AMPK interactions, resulting in ULK1 inactivation and autophagy shutdown. In starvation state, AMPK is activated, TOR is inactivated, Atg13 is dephosphorylated, and the Atg1 complex is completed to initiate autophagy. When AMPK is activated, mTOR is inactivated. Activated AMPK catalyzes ULK1 phosphorylation at Ser317, Ser467, Ser555, and Ser574, thereby promoting autophagy. AMPK, adenosine monophosphate-activated protein kinase; TOR.

Autophagy is divided into four key steps: initiation, autophagosome formation, maturation, and degradation (Feng et al., 2014). Various related proteins are involved in this process, such as autophagy-related gene (Atg), microtubule-associated protein one light chain 3 (MAP1LC3) (Kanki and Klionsky, 2010). The hallmark of autophagy initiation is the formation of autophagosomes, and the initiation process is mainly dependent on the ULK1 complex. ULK1 is a molecule with serine/threonine kinase activity, located at 12q24.33 and containing 28 exons (Casas et al., 2004). Post-translational modifications of ULK1, such as phosphorylation (Di Rienzo et al., 2019) and ubiquitination (Raimondi et al., 2019), are key to autophagy induction (Di Rienzo et al., 2019; Raimondi et al., 2019). When nutrients are exhausted, or growth factors are deprived, ULK1 is dephosphorylated, leading to ULK1 activation and autophagocytosis (Fritzen et al., 2016). In addition, ULK1 can form a stable complex with autophagy-related proteins 13 (Atg13), 101 (Atg101), and FIP200 (RB1CC1), which are direct homologs of mammalian Atg17 (Morselli et al., 2011; Kim et al., 2018). The ULK1-Atg13-FIP200-Atg101 complex regulates autophagy initiation and termination in mammalian cells by detecting the nutritional status of the cells. However, ULK1 kinase activity is regulated by mTORc1 and adenosine monophosphate-activated protein kinase (AMPK) (Losier et al., 2019). Under nutrient-rich conditions, AMPK is inactivated, TOR is activated to bind to ULK1 and inactivate ULK1, Atg13 is phosphorylated, the kinase activity of the Atg1 complex is reduced, and autophagy is inhibited (Fritzen et al., 2016). When nutrients are depleted, AMPK is activated, and mTOR is inactivated, activating ULK1 phosphorylation at Ser317, Ser467, Ser555, Ser574, Ser637, and Ser777, leading to activation of ULK1 and autophagy (Bujak et al., 2015) (Figure 3).

Before the formation of complete autophagosomes, there are two initial processes nucleation and elongation. In the nucleation stage, Beclin-1, vacuolin classification complex 34, phosphoinositol 3-kinase regulatory subunit 4 (ATG14), and other related proteins are activated to form the Beclin1-Atg14L-VPS15-VPS34 complex, which includes the catalytic core region and promotes phagocyte nucleation (Inoki et al., 2002; Mizushima, 2007). Next, the class III phosphoinositol 3‐kinase (PI3K) complex is required to form a mature autophagosome, which consists of five subunits (Atg14L, Beclin 1, VSP34, and VSP15) (Mizushima et al., 2001; Mizushima et al., 2003). In addition, the Atg5/Atg12/Atg16L complex, together with the lipid MAP1LC3, stimulates the extension of phagocytes, resulting in the formation of mature autophagosomes (Kabeya, 2000; Mizushima et al., 2001).

Once the autophagosome is formed, it transports its cargo to the lysosome in mammalian cells. When it reaches its destination, the outer membrane of the autophagosome fuses with the lysosomal membrane. Autophagosomes and lysosomes usually fuse to form autophagosomes under the action of cytoskeletal components (such as actin filaments and microtubules) (Monastyrska et al., 2009; Yang and Klionsky, 2009). A large number of functional proteins are involved in the formation of the autophagosomes, such as vesicle-associated membrane protein 3 (VAMP-3), sensitive factor attachment protein receptor (SNARE), and Syntaxin 17 (STX17) (Furuta et al., 2010; Itakura et al., 2012). After exposure to the acidic lumen and permanent lysosomal hydrolases, the inner membrane of the autophagosome and subsequent autophagy substances are degraded, and the components are exported back for use to the cytoplasm through lysosomal permease or are directed to the cell energy generation in the biosynthesis process (Yorimitsu and Klionsky, 2005).

### 3.3 The Regulatory Mechanism of Autophagy

**FIGURE 4:**
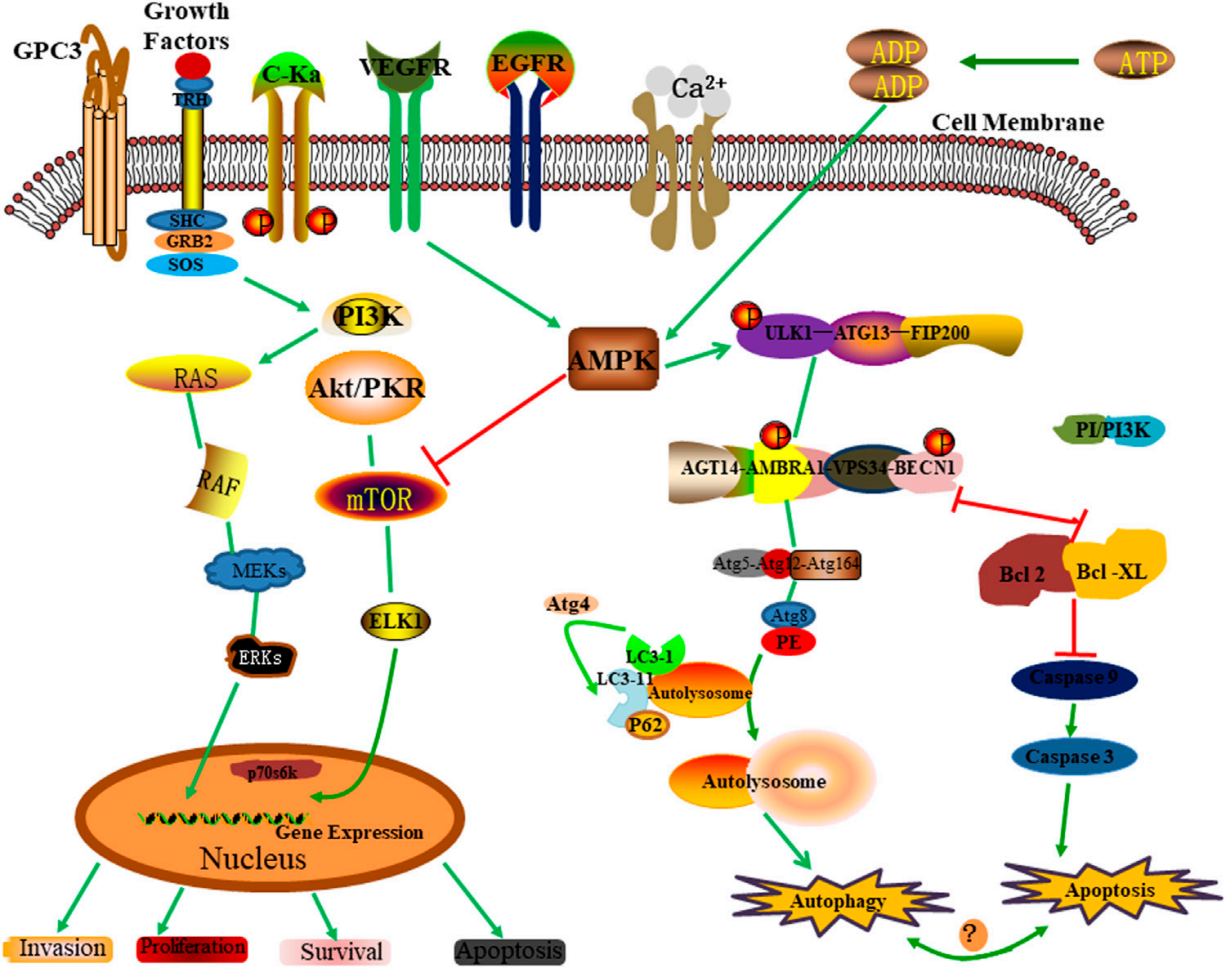
Autophagy regulatory mechanism.

Regulation is the key to specifically “turning on” autophagy for a required limited time. Autophagy is regulated by various factors, including several signaling molecules and cascades that regulate autophagy in response to many cellular and environmental cues (Meijer and Codogno, 2009; Rubinsztein et al., 2012b). These regulatory factors include mainly positive regulation of the serine/threonine-protein kinase ULK1 complex, Beclin1 complex, AMPK, and negative regulation of mTOR targets. In the previous section, we introduced the regulatory mechanism of the serine/threonine-protein kinase ULK1 complex in the process of autophagy. Next, we will focus on the positive regulation of the Beclin1 complex, AMPK, and the negative regulation of mTOR targets (Figure 4).

#### 3.3.1 Beclin1 Complex Pathway

Beclin1, a homologous protein of Atg6/VPS30 in yeast, interacts with class III PI3K (also known as VPS34) and plays an important role in forming autophagosomes, a key step in the autophagy process. Activation of the kinase activity of the Beclin 1-VPS34 complex promotes the production of phosphatidylinositol 3-phosphate, which promotes lipid membrane elongation, cargo recruitment, and autophagosome maturation (Kihara et al., 2001). Beclin 1 and VPS34 form two different complexes: In the Beclin1-VPS34 complex I, Atg14L is cross-linked between Beclin1 and VPS34-P150 complex, which mediates the formation of autophagosomes. In contrast, the Beclin 1-VPS34 complex II (Beclin 1 and VPS34-P150 complex bridged by UVRAG) regulates the vacuolin-sorting pathway (Itakura et al., 2008). Thus, Beclin 1-Vps34 complex I is one of the most important platforms for regulating autophagy activity through various cellular stimuli (Levine et al., 2015). The kinase activity of Beclin 1-VPS34 complex I is regulated either by post-translational modification or by direct binding to other proteins, which cause Beclin 1 to separate from specific subcellular sites, such as the Golgi body or cytoskeleton, or alter its interactions with Beclin 1-VPS34 complex I binding partners, including Atg5, ATG14 and Beclin-2 family proteins (Funderburk et al., 2010).

#### 3.3.2 AMPK Signaling

As a cellular energy sensor, the AMPK pathway plays an important role in regulating autophagy initiation (Bujak et al., 2015). AMPK, a major regulator of cell and organism metabolism in eukaryotes, is activated when intracellular ATP content is reduced (Mihaylova and Shaw, 2011). Thus, AMPK plays a key role in regulating growth and reprogramming metabolism as a positive regulator of autophagy (Gong and Zhang, 2021). AMPK regulates autophagy in the body in two ways. First, AMPK promotes autophagy by phosphorylating ULK1 and activating it (Egan et al., 2011; Kim et al., 2011). Second, AMPK may activate or promote autophagy by inhibiting the activity of mTORC1 through the TSC1/2-rhEb pathway (Inoki et al., 2003; Alers et al., 2012). When nutrition is sufficient, AMPK is inactivated, and mTOR binds to ULK1 Ser757, inhibiting the interaction between ULK1-AMPK, resulting in ULK1 inactivation and inhibition of autophagy. However, when the cells are hungry, AMPK is activated, mTOR is inactivated, and activated AMPK catalyzes ULK1 phosphorylation at Ser317, Ser467, Ser555, Ser574, Ser637, and Ser777, thereby promoting autophagy. Studies report that activating the AMPK/mTOR pathway may enhance autophagy and accelerate apoptosis in human neuronal cells (Meng et al., 2018).

#### 3.3.3 mTOR Signaling

Studies report that the mTOR signaling pathway can regulate autophagy and apoptosis in neurodegenerative diseases. mTOR is a negative regulator and a gated molecule of autophagy (Kim et al., 2011). It is composed of mTORC1 and mTORC2, which bind to ULK1’s serine 757 and inhibit AMPK-ULK1 interactions, resulting in ULK1 inactivation and inhibition of autophagy. In addition, it reacts to a variety of upstream signals (such as class I PI3K, IGF-1/2, MAPK) to inhibit autophagy (Stamenkovic et al., 2019). A recent study reported that metformin could act as a neuroprotective agent by inhibiting the mTOR signaling pathway after SCI to promote autophagy and inhibit apoptosis (Guo et al., 2018). In addition, a study by Lin et al. (2020) confirmed that zinc promotes autophagy by inhibiting mTOR activity (mTOR phosphorylation) by activating the AMPK signaling pathway, thus having a phyletic neuroprotective effect. In addition to the above studies, Gao et al. (2020), while working with SCI *in vitro* and *in vivo* experiments, found that Wnt-3a significantly activated autophagy of neurons after SCI by inhibiting the mTOR signaling pathway, thus playing a neuroprotective role.

## 4 Autophagy and Spinal Cord Injury

Kanno et al. first reported the overexpression of Beclin1 and LC3II autophagy proteins in experimental SCI lesions (Kanno et al., 2009a). Since then, many studies have focused on the autophagy mechanism and its influence on the SCI pathological process (Liu et al., 2015a). However, the autophagy role in SCI remains controversial. Most recent studies show that autophagy may protect and promote neurons recovery after SCI, while other research reports that autophagy can accelerate the progression of SCI.

### 4.1 Changes in Autophagy Levels in Spinal Cord Injury

Increased autophagy biomarkers (Beclin-1, LC3B II, and P63) levels and accumulation of autophagosomes were observed during the acute phase of secondary injury (Kanno et al., 19762011; Lai et al., 2008). Kanno et al. were the first to demonstrate the autophagy activation after SCI. Their experimental results showed significantly increased Beclin1 expression at the damaged site in a mouse model of a half-cut spinal cord. Beclin1 expression increased at 4 h after injury, peaked on the third day, and persisting still high on the 21st day (Kanno et al., 2009b). Hou et al. (2014) tested the autophagy activity in a rat model of spinal cord semi-incision and found the same result. Hao et al. (2013) detected Beclin1 and LC3 at mRNA and protein levels using a model of spinal cord contusion caused by a heavy-body blow. They determined that the autophagy level increased at 1 h after injury, peaked at 2 h, and returned to the normal level 72 h later. However, Wang et al. (2014a) used sterile blades to cut and scrape primary spinal cord neurons cultured *in vitro* to establish a cell model of SCI *in vitro*. The results showed that autophagy began to increase at 0.5 h after SCI, reached a peak at 24 h, and remained at a high level at 72 h after the injury. In addition, recent studies in animals and humans have also shown that autophagy biomarkers persist for weeks and months after primary trauma (Chen et al., 2014; Sakai et al., 2014). However, A recent study on quantitative analysis of single cells in complete sections of the spinal cord in the subacute phase (7 days after injury) of mouse contusion SCI showed higher LC3 staining in neurons in all injured sections than in uninjured sections. Therefore, it is considered that the abundance of autophagy generally increases in the subacute phase (7 days after injury) of contusion SCI in mice (Muñoz-Galdeano et al., 2018). The above results indicate that autophagy is significantly activated in the early stage after SCI in both animal and cell experiments, and the level of autophagy in SCI also varies according to the time elapsed after surgery. It also revealed the correlation between autophagy and SCI process. Therefore, determining how autophagy changes in the post-traumatic process and how it is expressed in relevant cells expressing cellular heterogeneity is key to establishing autophagy as a therapeutic target for SCI.

### 4.2 Autophagy Flux in Spinal Cord Injury

Autophagy flux is a process of formation, fusion, and degradation of autophagosomes. Recently, it has been reported that the disruption of autophagy flux after traumatic SCI is related to the induction of endoplasmic reticulum stress and motor neuron apoptosis (Liu et al., 2015b). However, the difference in autophagy flux may be related to various factors. Some studies have proposed that injury model or severity may differentially affect autophagy activation and the ability of autophagy flux to proceed to completion (Lipinski et al., 2015). Increased accumulation of autophagy markers LC3-II and SQSTM1 was observed in different SCI models after injury (Kanno et al., 19762011; Tanabe et al., 2011; Hou et al., 2013). Autophagy flux was found to be inhibited to varying degrees in various injury models, such as the acute contusion SCI model in rats and mice (Liu et al., 2015b), chronic spinal cord compression model in mice and rats (Tanabe et al., 2011; Chen et al., 2014), and spinal nerve ligation model in rats with neuropathic pain (Berliocchi et al., 2011). In addition, Verma et al. classified preclinical animal models of traumatic SCI into mild, moderate, and severe injury models. They found that in various injury models, the degree of injury may also differentially regulate the activation of autophagy and autophagy flux. A relatively mild invasive SCI can enable upstream activation of autophagy and maintenance of autophagy flux to provide neuroprotection (Lipinski et al., 2015; Verma et al., 2019). However, a relatively severe primary injury may lead to the over-activation of autophagy flux, leading to the death of autophagy neurons in the process of traumatic secondary SCI (Hao et al., 2013; Lipinski et al., 2015). In addition, other studies have suggested that autophagy flux progresses differently in different types of SCI (Luo and Tao, 2020). Autophagy flux is blocked in severe contusion injury but increases in hemisection injury. In compression injury, autophagic flux is inhibited after severe compression (15 g for 1 min) and enhanced after relatively moderate compression (30 g for 1 min) (Zhou et al., 2017b). The specific mechanism of autophagy flux change is not clear at present, but it may be due to intracellular Ca^2+^ overload in acute traumatic SCI causes lysosomal rupture and autophagy flux disruption due to calpain and calpain-mediated cleavage and inhibition of chaperone heat shock protein 70 (HSP70). This leads to the death of neurons and oligodendrocytes and an increase in neurological dysfunction in traumatic SCI (Ray et al., 2003; Yamashima and Oikawa, 2009). In addition, Liu et al. (2015a) reported that the accumulation of autophagosomes after SCI was not due to the enhancement of autophagy initiation but due to the inhibition of autophagy flux. Based on the above studies, it is not difficult to see that the change of autophagy flux after SCI is affected by many factors, such as the severity of injury type. It also reflects the disruption of lysosomal function after SCI. Suggesting that the autophagy disruption after SCI may aggravate endoplasmic reticulum stress and neuronal cell death.

### 4.3 Autophagy and Apoptosis in Spinal Cord Injury

Apoptosis is known to play a crucial role in SCI-induced axis breakage and cell death. Therapeutic regimens targeting anti-apoptotic mechanisms provide better neurological outcomes in experimental SCI (Luo and Tao, 2020). A large number of studies have reported a close relationship between autophagy and apoptosis. Many common signal transduction pathways, such as p53 protein, Bcl-2-homology-3-only (BH3-only) protein, and serine/threonine kinase, can affect autophagy and apoptosis. These processes suggest that the relationship between autophagy and apoptosis can be regulated by inhibiting each other’s cross. Activates c-Jun N-terminal protein kinase 1 (JNK1) in the context of injury and starvation, which phosphorylates the regulatory loop of Bcl-2 and then shuts down the interaction between Bcl-2 and Beclin-1. Isolated Beclin-1 promotes autophagy by activating core autophagy components, such as Vps34 and the Beclin-1/VPS34/VPS15 core complex. Phosphorylated Bcl-2 interacts with Bcl-2-Associated X Protein (Bax) to maintain the integrity of the mitochondrial membrane and promote anti-apoptotic function (Wei et al., 2008). Zhao et al. (Zhao et al., 2017) found that the expression of SIRT1, p-AMPK, Beclin-1, LC3-B, and Bcl-2 in mice with SCI increased in resveratrol-treated mice, while P62, caspase-3, caspase-9, and Bax were inhibited; resveratrol can play a neuroprotective role by activating autophagy and inhibiting apoptotic pathways *via* the AMPK signaling pathway. Furthermore, the production and maintenance of autophagy during the acute phase of traumatic SCI can promote neuroprotection. However, excessive hypoxia or nutritional stress can cause excessive autophagy or activate apoptosis and other cell death pathways. Therefore, we should focus on maintaining the balance between autophagy and apoptosis.

### 4.4 Autophagy Improves Spinal Cord Injury

A large number of studies have reported that the maintenance of appropriate autophagy flux in acute traumatic SCI can clear damaged cellular components, organelles, and cells and promote neuroprotection and functional recovery. Rapamycin is an inhibitor of the Ser/Thr kinase mTOR signaling pathway, which negatively regulates autophagy. It has recently been shown to promote autophagy by upregulating Beclin-1 and LC3b II, reducing apoptosis-induced neuron loss and significantly increasing motor function in rats with acute traumatic SCI (Wang et al., 2014b). In addition, in traumatic SCI, autophagy promotes beneficial opportunities for intracellular content degradation and circulation, thus enabling neurons to survive in an environment deficient in nutritional factors (Zhang et al., 2018). A recent study reported that curcumin treatment of traumatic SCI in rats promoted autophagy, prevented neuronal apoptosis, improved spinal cord integrity, and inhibited inflammatory response, thereby promoting functional recovery by inhibiting the Akt/mTOR signaling pathway (Li et al., 2021). In addition, some studies have found that appropriate activation of autophagy can maintain permeability after traumatic SCI-induced blood-brain barrier disruption. As a result, blood cell infiltration, inflammatory response, and neuronal cell death could be reduced, and neuronal function recovery promoted (Zhou et al., 2016).

## 5 The Dual Role of Autophagy in Spinal Cord Injury

Some scholars believe that the activation of autophagy is a protective feedback mechanism after SCI, while others find that the upregulation of autophagy can delay functional repair after SCI. Thus, the role of autophagy in SCI is “a double-edged sword,” according to many studies.

### 5.1 The Protective Effect of Induced Autophagy in Spinal Cord Injury

#### 5.1.1 Promotes Neuroprotection and Functional Recovery

Autophagy is a homeostasis mechanism of healthy cells and a cellular protection process against CNS diseases and injury (Nixon and Yang, 2012). Autophagy can be considered a survival mechanism because it restricts nerve cell death and promotes neuroprotective effects in traumatic SCI (Lipinski et al., 2015). In the central nervous system, autophagy fights neurodegeneration by clearing damaged proteins, organelles, and cells. Moreover, it plays an important role in self-digestion to maintain normal neurological function (Mizushima et al., 2008). There are two major intracellular protein degradation systems in autophagy, the ubiquitin-proteasome system (UPS) and the autophagy-lysosome system, which cooperate with each other in neuroprotective functions (Nedelsky et al., 2008). In animal experiments, Wu et al. (2018) found that the optimal dose of specific autophagy-inducing peptide Tat-Bec could induce neuronal autophagy and promote axonal growth in neurons exposed to the inhibitory substrate myelin reduce axonal contraction, thereby improving motor capacity after SCI (Williams et al., 2014). In addition, Goldshmit et al. (2015) reported that rapamycin could effectively increase LC3 and Beclin1 levels after SCI by activating autophagy and Akt signaling pathways, inhibiting cell apoptosis, and promoting functional recovery. Atorvastatin is a lipid-lowering drug with neuroprotective effects. Studies have shown that atorvastatin can activate autophagy in rats with traumatic SCI (increase autophagy-related proteins Beclin-1 and LC3b II), inhibit apoptosis, and thus promote the recovery of neurological functions (Gao et al., 2016). A recent study showed that the expression of autophagy biomarkers Beclin-1 and LC3B II increased after the injection of VEGF165 in young male Wistar rats with induced traumatic SCI. It also improved Basso-Beattie-Bresnahan (BBB) scale scores for motor neuron loss (Wang et al., 2015). In addition, a study by Zhao et al. found that resveratrol could promote autophagy through the AMPK/SIRT1 signaling pathway, providing functional neuroprotection in traumatic SCI (Luo and Tao, 2020). The reports we referred to demonstrate that autophagy plays an important role in nerve protection and functional recovery after SCI.

#### 5.1.2 Reduced Neuronal Cell Apoptosis

Neuron apoptosis is the main pathological feature of SCI, and autophagy can improve neuronal injury by inhibiting apoptosis. The autophagy and apoptosis processes in SCI have been extensively studied. From cell survival to cell death, autophagy can significantly control the pathological process in traumatic SCI. Among nerve cells, neurons are most likely to activate autophagy to eliminate toxic proteins and damaged mitochondria (mitochondria) and inhibit apoptosis from promoting neuroprotective effects in traumatic SCI (Wang et al., 2018a). In addition, autophagy may regulate apoptosis by actively degrading pro-apoptotic proteins and organelles (such as caspase-8 and mitochondria). In an animal study, Ren et al. (Ren et al., 2019) found that family member 2 (TCTN 2) long non-coding RNA enhanced autophagy by targeting the miR-216b-beclin-1 pathway, thus improving neuronal apoptosis and alleviating SCI.

#### 5.1.3 Inhibition of Inflammation

**FIGURE 5:**
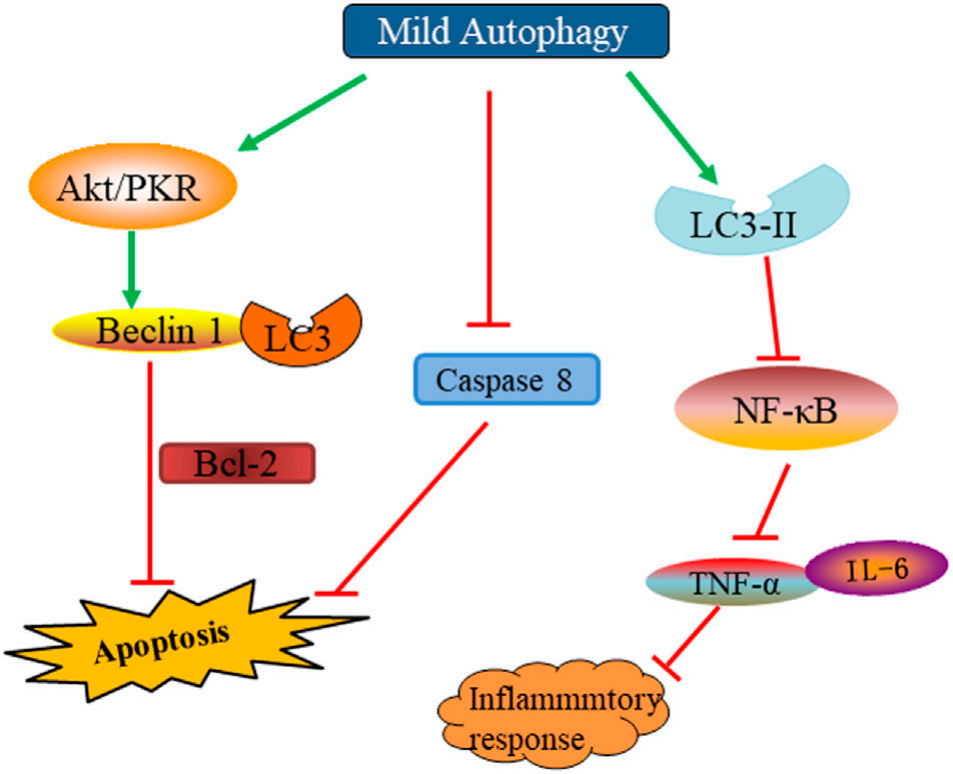
The beneficial effect and mechanism of moderate autophagy on SCI.

Autophagy plays an important role in the maintenance of homeostasis. Recent studies have found that the activation of autophagy can inhibit the inflammatory response in SCI. A complex series of molecular and cellular events, including inflammatory responses, occurs after SCI. Inflammatory reactions may lead to secondary tissue damage and further to functional loss (David et al., 2012). Therefore, reducing inflammation after SCI is crucial for nerve recovery. Research has shown that autophagy could inhibit the body’s inflammatory response and reduce the inflammatory damage caused to tissues by regulating the activation of the NOD-, LRR- and pyrin domain-containing protein three inflammasome and the clearance of mitochondrial reactive oxygen species (Cao et al., 2019). In SCI, the down-regulated nuclear factor kappa light chain enhancer (NF-κB) pathway has been shown to inhibit lipopolysaccharide-induced inflammation by activating autophagy. *In vitro* knockout of p65 to inhibit NF-κB pathway showed up-regulation of autophagy markers LC3-II, down-regulation of p62, down-regulation of tumor necrosis factor (TNF)-α and interleukin (IL)-1. However, the autophagy inhibitor chloroquine significantly upregulated TNF-α and IL-1, inhibited the ubiquitinated nuclear factor of kappa light polypeptide gene enhancer in B-cells inhibitor, alpha (IκBα) degradation, blocked the nuclear translocation of nuclear factor κBp65 and the expression of inflammatory factors. p62 overexpression increases IκBα degradation and improves inflammatory responses (Wu et al., 2018). Wang et al. reported direct inoculation of VEGF165 in rat models with trauma-induced SCI, which showed decreased levels of inflammatory cytokines, such as IL-1β, TNF-α, and IL-10 (Wang et al., 2015) (Figure 5) (Table 1).

**TABLE 1:** The beneficial effects of autophagy on SCI.

Functions	Mechanism	References
Promotes neuroprotection and functional recovery	Activation of AKT pathway promotes the expression of LC3 and Beclin 1, inhibits apoptosis and promotes functional recovery	Nedelsky et al. (2008), Nixon and Yang (2012)
Reduced neuronal cell apoptosis	Degradation of pro-apoptotic proteins and organelles (e.g., Caspase-8, mitochondria)	Williams et al. (2014)
Inhibition of inflammation	Up-regulate the expression of LC3-II, decrease the activity of NF-κB pathway, to down-regulate the expressionof TNF-α and IL-1	Li et al. (2021)

### 5.2 The Deleterious Role of Autophagy in Spinal Cord Injury

**FIGURE 6:**
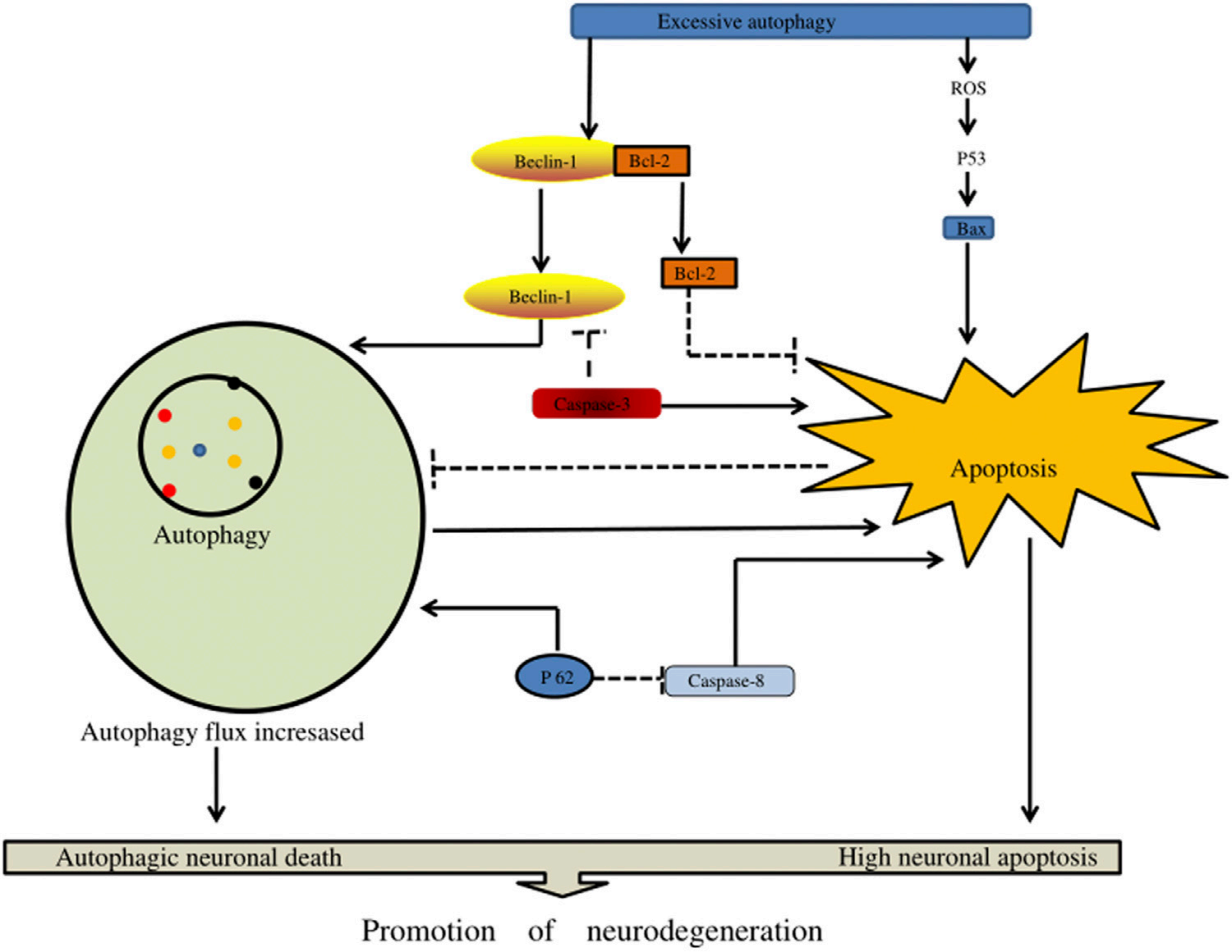
The effect of excessive autophagy on spinal cord injury. Excessive autophagy leads to the separation of Beclin-1 and Bcl-2 and the production of excessive reactive oxygen species in P53 signaling, resulting in the generation of free Bax. In addition, Beclin-1 and P62 can enhance autophagy flux, resulting in autophagy death of nerve cells, and enhance apoptotic factors (such as caspase-3, caspase-8, and Bax) to accelerate nerve cell apoptosis.

In the pathogenesis of severe traumatic SCI, an increase in autophagy flux may contribute to neuronal cell death through excessive autophagy (autophagy death) and/or through the activation of apoptosis and other cell death mechanisms. Excessive autophagy enhances caspase-1 activation through a nonclassical Atg5-dependent pathway, promoting inflammasome activation and increasing IL-1β and IL-18 synthesis. In addition, it could promote the secretion of these pro-inflammatory factors into the cytoplasm, aggravate the inflammatory damage of tissues, and lead to autophagic cell death (Dupont et al., 2011). Chen et al. (Chen et al., 2012) reported research in animals that determined that the body activates autophagy when SCI occurs. Excessive autophagy and autophagy may lead to autophagic death of neurons, thus affecting the regeneration of axons and the recovery of body neural function (Figure 6) (Table 2).

**TABLE 2:** The harmful effects of autophagy on SCI.

Functions	Mechanism	References
Promotes apoptosis	Excessive autophagy induces self-digestion of nerve cells and promotes cell apoptosis	Wang et al. (2015)
Promotes inflammatory response	Activation of caspase-1 promotes the activation of inflammatory questions, thereby increasing the synthesis of IL-1β and IL-18	Wang et al. (2015), Wang et al. (2018a)

## 6 Targeted Autophagy Therapy in Spinal Cord Injury

Current treatment for spinal cord injury (SCI) include conservative, surgical, and combined therapies that are supportive at best, the lack of better treatment solutions looms large on neurological science and medicine. SCI is a substantial health epidemic throughout the world, and the relative stagnation of developments past corticosteroid therapies necessitates other routes of improving function and quality of life in these patients (McDonald and Sadowsky, 2002). There are several methods being utilized as experimental therapies for patients with SCI, including electrical nerve stimulation (Keith et al., 1996), therapeutic hypothermia (Wang and Pearse, 2015), and cell therapy (McDonald, 1999). However, the results of this treatment are always unsatisfactory, and a series of complications unintentionally arise after the treatment, which causes patients to suffer again and face a second operation. Therefore, it is critical for the development of innovative therapeutic targets and therapies.

The activation of autophagy is an important event, and the promotion or inhibition of autophagy may be a promising therapeutic strategy to manage the pathogenesis of traumatic SCI. Currently, there are few clinical trials of targeted autophagy compounds and targeted autophagy for spinal cord injury, but based on the role of these compounds in preclinical studies in SCI, targeted autophagy is still an attractive treatment strategy for SCI (Table 3).

**TABLE 3:** Maincompounds directed towards autophagy for the treatment of SCI.

Compounds	Effect on autophagy	Functions	Function related proteins	Signaling pathway	References
Rapamycin	Activating	Promoted autophagy, reduced neuronal apoptosis, increased locomotor function and promoted neuronal survival and axonogenesis	Beclin-1, LC3B II	mTOR signaling pathway	Lai et al. (2008), Sekiguchi et al. (2012), Lipinski et al. (2015), Lin et al. (2016)
VEGF165	Activating	Reduces inflammatory factors, Reduce neuron loss, Promotes neuromotor function	Beclin-1, LC3B II	**—**	Wang et al. (2015)
Retinoic acid	Activating	Improves neurological recovery	LC3B II, p62	**—**	Zhou et al. (2016)
Atorvastatin	Activating	Inhibition of neuronal apoptosis Promotethe recovery of nerve function	Beclin-1, LC3B -II,caspase-9	**—**	Goldshmit et al. (2015)
Resveratrol	Activating	Inhibition of neuronal apoptosis, Promotethe recovery of nerve function	Beclin-1, LC3B II	AMPK/SIRT1 signaling pathway	Gao et al. (2016)
Curcumin	Activating	Inhibitory nerve cell apoptosis, Maintain spinal cord integrity	**—**	Akt/mTOR signaling pathway	Li et al. (2021)
VPA	Inhibiting	Reduces neuronal autophagy death, Promote the recovery of nerve function	**—**	**—**	Hao et al. (2013)
E2	Inhibiting	Inhibits excessive autophagy, reduces neuronal cell death	Beclin-1, LC3B II	**—**	Zhang et al. (2017)
3-MA	Inhibiting	Promotes the survival of distal, red myeloid neurons	**—**	PI3K signaling Pathway	Bisicchia et al. (2017)
Ginsenoside Rb1	Inhibiting	Inhibits excessive autophagy, reduces the death of nerve cells, promotes the recovery of nerve function	Beclin-1, LC3B II	**—**	Wang et al. (2018b)

### 6.1 Factors Increasing Autophagy (Autophagic Agonists)

Studies of animal models have shown that promoting autophagy is crucial for protecting CNS cells and promoting motor function recovery in traumatic SCI. In traumatic SCI, autophagy promotes the degradation and circulation of cellular contents, allowing neurons to survive in an environment deficient in trophic factors (Zhang et al., 2018). Current studies on autophagy in SCI view targeting mTOR and AMPK pathways in traumatic SCI as a promising treatment method (Thellung et al., 2019). Zhang et al. (2018), Thellung et al. (2019) confirmed that selective RNAi-mediated mTORc1 suppression and pharmacological mTORC1 inhibition could prevent the progression of SCI by inducing autophagy *in vivo* and *in vitro*. Rapamycin is an inhibitor of the mTOR signaling pathway of Ser/Thr kinase. Goldshmit et al. (Goldshmit et al., 2015) reported that a single injection of rapamycin eliminated p62/sqSTM1-induced active autophagy flux, inhibited mTORc1 downstream effect on p70S6K, and reduced macrophage/neutrophil infiltration into the lesion site. It also prevented microglial activation and inflammation, blocked astrocyte proliferation, increased p-Akt levels, and promoted neuronal survival and axon generation in the injured site after traumatic SCI in mice. Zhang et al. (2017) also found metformin treatment-induced activation of AMPK and inhibition of its downstream mTOR signaling pathway in rats *in vivo* and *in vitro*, suggesting that metformin improved functional recovery from traumatic SCI in rats and reduced nerve cells apoptosis. In addition, a recent study reported that curcumin promoted autophagy in the treatment of traumatic SCI in rats. Furthermore, it prevented neuronal apoptosis, improved spinal cord integrity, produced remyelination, inhibited inflammatory response, and thus promoted functional recovery by inhibiting the Akt/mTOR signaling pathway (Li et al., 2021). Zhao et al. (2017) identified a neuroprotective role of resveratrol in traumatic SCI and identified a potential relationship between AMPK/SIRT1 signaling, autophagy, and apoptosis. Resveratrol promotes autophagy and inhibits apoptosis through AMPK/SIRT1 signaling *in vivo* in traumatic spinal cord injury. In conclusion, pharmacological activation of autophagy in traumatic SCI is a promising approach for neuroprotection.

### 6.2 Inhibitory Autophagy (Autophagy Antagonists)

Some researchers believe that autophagy plays an important role in the neurodegeneration and the pathogenesis of various central nervous system diseases, traumatic brain, and spinal cord injury. When autophagy potentiates neurodegeneration in traumatic SCI, the therapeutic inhibition of autophagy is arguably an attractive alternative treatment strategy for neuroprotection and functional recovery. Several studies have demonstrated that the use of antagonists, e.g., valproic acid (VPA), 3-methyladenine (3-MA), and 17β-estradiol (E2), inhibits autophagy to prevent nerve tissue damage and improve neurological function in experimental animal models with different traumatic SCI. VPA is a known neuroprotective agent that can significantly reduce autophagy biomarkers after injury. Treatment with VPA after an injury can improve BBB scores after traumatic SCI, reduce autophagy death and myelin injury, increase the number of anterior horn motor neurons, and promote motor function recovery (Hao et al., 2013). A recent study suggested that 17β-estradiol (E2) could decrease the expression of autophagy-related proteins, such as Beclin-1 and LC3b II, in traumatic SCI, thereby improving the motor function and reducing the loss of motor neurons by inhibiting the occurrence of autophagy (Lin et al., 2016). In addition, other studies have found that 3-methyladenine is an inhibitor of autophagy, which blocks the formation of autophagosomes (APs) by inhibiting class III PI3K, thus significantly reducing distal neurodegeneration and improving spontaneous functional recovery (Bisicchia et al., 2017).

## 7 Discussion

Recently, an increasing number of studies have shown that autophagy plays a crucial role in the treatment and progression of SCI. Autophagy activation is an important event in traumatic SCI. Promotion or inhibition of autophagy may be a promising therapeutic strategy to manage the pathogenesis of traumatic SCI. However, the exact role of autophagy in SCI is controversial, and most studies show that the activation of autophagy could alleviate SCI and contribute to the recovery of nerve function. On the other hand, relatively few studies report that excessive activation of autophagy could lead to the death of nerve cells. Therefore, there is an urgent need to explore further the function and mechanism of autophagy activation in the field of SCI. Although current studies on autophagy and SCI are relatively extensive, some issues still need to be deeply considered. First, how to evaluate whether the activation of autophagy is favorable or unfavorable at different levels of SCI? Future studies should explore further the molecular mechanism of autophagy signal transduction. A better understanding of the relationship between autophagy and SCI would provide a clear theoretical basis for the treatment of SCI. Second, studies on the use of autophagy as a therapeutic target of SCI are still being conducted *in vitro* and in animal models, and there is little clinical evidence for the application of autophagy inhibitors and autophagy activators. Therefore, further clinical studies are needed to determine the efficacy and safety of targeted autophagy therapy for SCI. In conclusion, although autophagy plays a double-sided role in SCI, the activation or inhibition of autophagy may be a potential therapeutic target for the treatment of SCI.
